# *AR* splice variants in circulating tumor cells of patients with castration‐resistant prostate cancer: relation with outcome to cabazitaxel

**DOI:** 10.1002/1878-0261.12529

**Published:** 2019-06-28

**Authors:** Anieta M. Sieuwerts, Wendy Onstenk, Jaco Kraan, Corine M. Beaufort, Mai Van, Bram De Laere, Luc Y. Dirix, Paul Hamberg, Aart Beeker, Hielke J. Meulenbeld, Geert‐Jan Creemers, Wytske M. van Weerden, Guido W. Jenster, Annemieke J. M. Nieuweboer, Ron H. J. Mathijssen, Ronald de Wit, John W. M. Martens, Stefan Sleijfer

**Affiliations:** ^1^ Department of Medical Oncology Erasmus MC Cancer Institute Rotterdam The Netherlands; ^2^ Department of Medical Oncology Cancer Genomics Netherlands Rotterdam The Netherlands; ^3^ GZA Hospitals Sint‐Augustinus Wilrijk Belgium; ^4^ Center for Oncological Research University of Antwerp Antwerp Belgium; ^5^ Department of Internal Medicine Franciscus Gasthuis and Vlietland Rotterdam The Netherlands; ^6^ Department of Internal Medicine Spaarne Gasthuis Hoofddorp The Netherlands; ^7^ Department of Internal Medicine Gelre Ziekenhuizen Zutphen The Netherlands; ^8^ Department of Internal Medicine Catharina Ziekenhuis Eindhoven The Netherlands; ^9^ Department of Urology Erasmus MC Rotterdam The Netherlands

**Keywords:** androgen receptor, AR splice variants, cabazitaxel, castration‐resistant prostate cancer, circulating tumor cells

## Abstract

The androgen receptor splice variant (*AR‐V*) 7 in circulating tumor cells (CTCs) is a predictor for resistance to anti‐AR‐targeted treatment, but not to taxane‐based chemotherapy in metastatic castration‐resistant prostate cancer (mCRPC). In this study, we investigated whether the presence of two constitutively active variants (*AR‐V3, AR‐V7*) and two other conditionally activated variants (*AR‐V1, AR‐V9*) vs full‐length androgen receptor (*AR‐FL*) measured in CTCs from patients with mCRPC were associated with outcome to therapy with the taxane cabazitaxel. Blood was collected at baseline and after two cycles of cabazitaxel from 118 mCRPC patients starting cabazitaxel in a prospective phase II trial. CellSearch‐enriched CTCs were enumerated and in parallel characterized for the presence of the *AR‐V*s by reverse transcription quantitative polymerase chain reaction. Correlations with CTC and prostate‐specific antigen response to cabazitaxel as well as associations with overall survival (OS) were investigated. All *AR‐V*s were frequently present and co‐expressed at frequencies of 31–48% at baseline and at 19–40% after two cycles of cabazitaxel. No specific directions of change in the measured variants were detected between the start of treatment and after two cycles of cabazitaxel. No associations between the presence of *AR‐V3* and *AR‐V7* and outcome to cabazitaxel were observed. While a reduction in CTCs to < 5 CTCs during treatment (CTC5‐response) was less often observed in patients with *AR‐V9*‐positive CTCs at baseline (*P* = 0.004), the CTC5‐adjusted detection of *AR‐V1* after two cycles of cabazitaxel was an independent prognostic factor for OS [HR 2.4 (95% CI 1.1–5.1, *P* = 0.03)]. These novel findings are expected to contribute to more personalized treatment approaches in mCRPC patients.

**Abbreviations**
AR: androgen receptor
AR‐FL: full‐length androgen receptor
AR‐V(1/3/7/9): androgen receptor splice variant (1/3/7/9; )
cDNA: complementary deoxyribonucleic acid
*C*_q_: cycle threshold for quantification
CRPC: castration-resistance prostate cancer
CTC: circulating tumor cell
HR: hazard ratio
IQR: interquartile range
LOD: limit of detection
LOQ: limit of quantification
mCRPC: metastatic castration‐resistant prostate cancer
mRNA: messenger ribonucleic acid
OS: overall survival
PSA: prostate‐specific antigen
RR: response rate
RT–qPCR: reverse transcription quantitative polymerase chain reaction
SD: standard deviation

## 1 Introduction

The presence of the androgen receptor (AR) splice variant (*AR‐V*) 7 in circulating tumor cells (CTCs) was recently shown to predict resistance to new‐generation anti‐AR‐targeted treatments (abiraterone acetate and enzalutamide), but not to taxane‐based chemotherapy in patients with metastatic castration‐resistant prostate cancer (mCRPC) (Antonarakis *et al.*, 2015; Antonarakis *et al.*, 2017; Antonarakis *et al.*, 2014; De Laere *et al.*, 2017; De Laere *et al.*, 2019; Nakazawa *et al.*, 2015; Onstenk *et al.*, 2016; Onstenk *et al.*, 2015; Scher *et al.*, 2018; Scher *et al.*, 2017; Scher *et al.*, 2016; Tagawa *et al.*, 2019). If further validated, like currently ongoing in the CABA‐V7 study (NCT03050866), the *AR‐V7* status of CTCs may be used in clinical care to select the best treatment for an individual patient at a specific time (Sieuwerts *et al.*, 2018).

Similar to the constitutively, ligand‐independent, active *AR‐V7*, several other *AR‐Vs*, including *AR‐V3* that lack the ligand‐binding domain, have been identified*.* In addition, more cell‐context‐dependent variants like *AR‐V1* and *AR‐V9*, which like the full‐length AR (*AR‐FL*) are conditionally activated variants, have been described (Hu *et al.*, 2009; Hu *et al.*, 2011; Jernberg *et al.*, 2017). The clinical relevance of these splice variants to predict the type of response to taxane‐based chemotherapy in patients with mCRPC, however, remains to be established (Antonarakis *et al.*, 2016; De Laere *et al.*, 2019).

In previous reports, we demonstrated in cases included in the prospective CABARESC trial (Nieuweboer *et al.*, 2017) the feasibility of measuring the *AR‐V7* status of CellSearch‐enriched CTCs (Sieuwerts *et al.*, 2018) and showed that the presence of the constitutively active *AR‐V7* variant at baseline had no association with outcome to cabazitaxel chemotherapy (Onstenk *et al.*, 2015). In the current study, we used our CellSearch‐based assay to further investigate the role of *AR‐V7* in CTCs in an extended series recruited within the framework of the prospective CABARESC trial (Nieuweboer *et al.*, 2017). Besides *AR‐V*7, we determined the prevalence of another constitutively active, ligand‐independent, AR variant (*AR‐V3*) and two, like *AR‐FL*, conditionally activated variants (*AR‐V1* and *AR‐V9*) in CTCs at baseline and after two cycles of cabazitaxel. Finally, we investigated their possible clinical relevance.

## 2 Patients and methods

### 2.1 Patients

As reported before (Onstenk *et al.*, 2016; Onstenk *et al.*, 2015), patients with progressive mCRPC were recruited from the multicenter prospective phase II CABARESC trial (Dutch trial registry number NTR2840) (Nieuweboer *et al.*, 2017). In our pilot study, we reported on 29 patients with ≥ 10 CTCs at baseline (Onstenk *et al.*, 2015). Here, we report on all patients from the trial from which we had collected CTCs. Full inclusion criteria are listed in Data S1. Patients were randomized between three‐weekly cabazitaxel, 25 mg·m^−2^ with or without budesonide, a locally active corticosteroid, during the first two treatment cycles. No difference in overall survival (OS) [log‐rank (Mantel–Cox) *P* = 0.41] or incidence or severity of cabazitaxel‐induced diarrhea (Nieuweboer *et al.*, 2017) was seen between the two arms, and therefore, the budesonide treatment arm was only evaluated as a stratification factor in the comparisons between the patient groups with CTCs negative or positive for the *AR‐Vs*. The work described has been carried out in accordance with Ethical Principles for Medical Research Involving Human Subjects (World Medical Association Declaration of Helsinki). The Erasmus MC and the local Institutional Review Boards at sites that collaborated in the study lent approval. All patients provided written informed consent.

### 2.2 Blood sampling and processing

Blood for the CTC analyses was sampled in CellSave [Menarini, Silicon Biosystems, Castel Maggiore (BO), Italy] and EDTA tubes before the first and after the second cycle of cabazitaxel. As described before (Onstenk *et al.*, 2015; Sieuwerts *et al.*, 2018), CTCs were enumerated and characterized from 7.5 mL peripheral CellSave and EDTA blood, respectively, using the CellSearch System (Menarini Silicon Biosystems). The CTC enumeration was performed in CellSave blood within 96 h after sampling and the characterization in EDTA blood within 24 h after sampling. CellSearch‐enriched CTCs from the EDTA characterization tube were subjected to RNA isolation using the AllPrep DNA/RNA Micro Kit (Qiagen, Hilden, Germany) followed by complementary deoxyribonucleic acid (cDNA) generation and preamplification of individual genes. Individual transcript expression levels were measured using quantitative polymerase chain reaction (qPCR) applying the same protocol as described before (Onstenk *et al.*, 2015; Sieuwerts *et al.*, 2018).

### 2.3 Validation processes of the qPCR assays

Details of the used TaqMan Gene Expression Assays (Applied Biosystems, Carlsbad, CA, USA) can be found in Table S1. Analytical validation data for each of the *AR‐V* measurements are given in Tables S2 toS4. 

In brief, all qPCR assays performed with an equal efficiency [slope log cDNA input vs cycle threshold for quantification (*C*_q_) value: −3.38 to −3.82]. The limit of detection (LOD), defined as the lowest input of RNA isolated from VCaP cells that could be reliably identified as being qualitatively present in the sample, was calculated to be the RNA equivalent of 0.03 (for *AR‐WT*) to 0.5 (for *AR‐V3*) VCaP cells in the final reverse transcription qPCR (RT–qPCR). The limit of quantification (LOQ), defined as the lowest input of RNA isolated from VCaP cells that could be reproducibly quantified, was set at a CV of less than 25% and calculated to be equal to the RNA equivalent of 0.02 (for *AR‐V1*) to 12 (for *AR‐V9*) VCaP cells in the final RT–qPCR (Table S2). For the intralaboratory validation, ddCq AR and its splice variants results were generated by five different technicians, starting at the RNA isolation step. No significant differences were observed between the data generated by the different technicians (Kruskal–Wallis test *P* > 0.05) (Table S3). For the interlaboratory validation, RNA extracted from five different cell lines and CTC fractions of 10 different patients with unknown *AR* status were exchanged between two laboratories (Rotterdam and Antwerp). For both the cell line and clinical samples, similar outcomes for *AR* and its splice variants were obtained (Table S4).

### 2.4 Normalization and statistical analysis

Evaluation of the RT–qPCR data was performed as described in full detail before (Sieuwerts *et al.*, 2018). In brief, only those samples with (a) an average *C*_q_ of the reference genes *GUSB, HMBS,* and *HPRT1* < 26.5 (indicating sufficient overall RNA quality and quantity) and (b) an average *C*_q_ of the epithelial genes *EPCAM* and *KRT19* < 26.0 (indicating sufficient epithelial tumor cell input) were selected for the analyses. All *C*_q_ values were normalized to the average *C*_q_ value of the two epithelial genes to avoid confounding of the analyses by the number of CTCs present in a sample. As established before for *AR‐V7 *(Sieuwerts *et al.*, 2018), samples were considered to be positive for any of the *AR* variants if a positive signal was detected within 8.5 cycles after the epithelial signal was detected. To ensure data were comparable in‐between experiments, a calibrator consisting of total RNA from cultured VCaP prostate cancer cells was included in each RT–qPCR session and processed identically to the samples. To calculate the calibrator normalized messenger ribonucleic acid (mRNA) expression level of the target, the delta–delta *C*_q_ (dd*C*_q_) method was used.

The primary endpoint of this study was the difference in CTC response rate (CTC‐RR), either defined as a conversion from ≥ 5 CTCs/7.5 mL to < 5 CTCs during treatment (CTC5) (de Bono *et al.*, 2008) or a clearance from ≥ 1 CTC (s) to 0 during treatment (CTC0) (Heller *et al.*, 2017), between patients with CTCs positive for one of the *AR‐Vs* and patients with CTCs negative for the particular *AR‐V* splice variant at baseline. Secondary objectives comprised prostate‐specific antigen (PSA)‐RR (30% and/or 50% decline from baseline to 12 weeks) and OS by the different *AR‐V*s. No formal sample size calculations were performed since we were restricted to the inclusion of patients from the CABARESC trial who consented for additional blood sampling for CTCs. Therefore, our analyses should be considered exploratory.

Standard statistical tests were applied to address the objectives. Differences between patient groups were tested by chi‐square or Fisher’s exact test. Survival was calculated from the registration date in the CABARESB trial, that is, start with cabazitaxel, until the date of death or last contact in the case of baseline parameters and from the time of second blood sampling until date of death or last contact for the on‐study parameters. The survival analyses were conducted using univariate and multivariate (with backward stepwise selection) Cox regression models. For the post‐treatment OS analyses, we conducted landmark analyses to compensate for the 6‐week treatment by removing the patients which experienced an event prior to the 6‐week landmark and then resetting the time to zero at the landmark. All statistical tests were two‐sided. Bootstrapping and a stringent *P* value < 0.01 were applied to correct for multiple testing. Statistics were performed using spss 24.0 (IBM Corporation, Armonk, NY, USA).

## 3 Results

### 3.1 Patient characteristics

**Figure 1:**
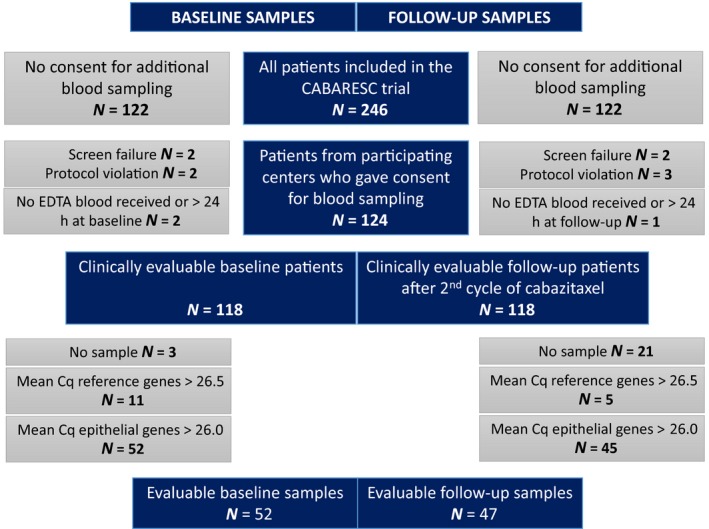
Selection of evaluable patients for the analyses.

For the current analyses, we selected all *n* = 124 patients that had been included in the prospective CABARESC trial (Nieuweboer *et al.*, 2017) and who had consented for additional blood sampling. Figure 1 shows the selection of patients and evaluable samples. Patient characteristics for the finally clinically evaluable *n* = 118 patients and *n* = 104 patients with an evaluable *AR* profile are summarized in Table 1 and specified for all *n* = 124 patients which gave informed consent for additional blood sampling in Table S5. All patients had been previously treated with docetaxel, and one‐third had also received next‐generation AR‐targeted agents (Table 1, left column). As expected, patients with sufficient epithelial cell input in the baseline blood sample, indicating the presence of CTCs in the sample (evaluable patients with epithelial signal), had worse baseline prognostic characteristics (higher LDH, AP, PSA and CTC count) than the patients with a low or absent epithelial signal in the blood sample (HBD‐like patients, Table 1, left columns).

**Table 1:** Patient characteristics. Patient characteristics of all patients with at least one available blood sample (*N *= 118 from the total of 124, first column) and the evaluable patients for the analyses with a baseline blood sample containing sufficient reference and epithelial gene signal (*N *= 52, patients with epithelial signal, third column) and non‐evaluable patients with insufficient epithelial signal, indicating the absence of tumor cell signal (*N *= 52, HBD‐like patients, second column); *P *values in the fourth column are from the comparisons between the evaluable and excluded patients. The fifth and sixth columns show the characteristics of evaluable patients with *AR‐V7* negative CTCs (*N *= 27) or *AR‐V7 *positive CTCs (*N *= 25) at baseline; *P *values in the last column are from the comparisons between the patients with *AR‐V7* positive and negative CTCs, respectively. All *P *values are from non‐parametric Mann‐Whitney U test (for age, baseline chemistry and CTC counts) and Fisher exact tests for the categorical variables.

	All patients		HBD‐like patients		Patients with epithelial signal		*P* value	*AR‐V7*‐negative patients		*AR‐V7*‐positive patients		*P* value
*N*	118	100%	52	100%	52	100%		27	100%	25	100%	
Mean age (years ± SD)	69 ± 7		71 ± 7		69 ± 7		0.17^a^	70 ± 8		68 ± 7		0.23^a^
WHO performance status												
0	60	51%	28	54%	26	50%	0.84^b^	13	48%	14	56%	1.00^b^
1	58	49%	24	46%	26	50%		14	52%	12	48%	
*N* prior chemotherapy lines												
1	112	95%	50	96%	48	92%	0.68^b^	27	100%	21	84%	**0.047** b
2	6	5%	2	4%	4	8%		0	0%	4	16%	
Prior antiandrogens												
None	75	64%	33	63%	34	65%		16	59%	18	72%	
Orteronel	23	19%	3	6%	6	12%	0.74^b^	5	19%	1	4%	0.19^b^
Abiraterone	8	7%	14	27%	7	13%	0.14^b^	2	7%	5	20%	0.24^b^
Enzalutamide	11	9%	1	2%	5	10%	0.44^b^	4	15%	1	4%	0.35^b^
Both orteronel and enzalutamide	1	1%	1	2%	0	0%		0	0%	0	0%	
With budesonide												
No	63	53%	25	48%	33	63%	0.17^b^	17	63%	16	64%	1.00^b^
Yes	55	47%	27	52%	19	37%		10	37%	9	36%	
Baseline chemistry												
Lactate dehydrogenase (median, IQR)	312 (216–454)		270 (200–372)		371 (250–575)		**0.002** a	335 (224–671)		396 (275–554)		0.89^a^
Alkaline phosphatase (median, IQR)	128 (84–240)		122 (79 −162)		174 (98–339)		**0.009** a	150 (68–368)		192 (127–310)		0.16^a^
Prostate‐specific antigen (median, IQR)	149 (49–365)		106 (35–298)		209 (72–510)		0.046^a^	186 (64–500)		232 (77–707)		0.46^a^
Circulating tumor cells	*N *= 112		*N *= 51		*N *= 50			*N *= 26		*N *= 24		
Median (IQR)	16 (3–95)		7 (1–19)		83 (8–230)		**< 0.0001** a	48 (5–230)		98 (35–243)		0.46^a^
CTCs > 0	97	87%	39	76%	49	98%	0.59^b^	26	100%	23	96%	0.48^b^
CTCs ≥ 5	76	68%	28	55%	40	80%	**0.010** b	20	77%	20	83%	0.73^b^
Cycles of cabazitaxel received (median, IQR)	7 (4–9)		8 (4–10)		6 (3–8)			6 (3–8)		6 (3–8)		
Abiraterone/enzalutamide after cabazitaxel												
No	47	40%	22	42%	22	42%		13	48%	9	36%	
Abiraterone	28	24%	10	19%	13	25%	0.80^b^	8	30%	5	20%	1.00^b^
Enzalutamide	33	28%	15	29%	13	25%	0.81^b^	3	11%	10	40%	0.08^b^
Both	9	8%	5	10%	4	8%	1.00^b^	3	11%	1	4%	0.64^b^
Follow‐up CTC count	*N *= 100		*N *= 42		*N *= 45			*N *= 23		*N *= 22		
Interval between start of cabazitaxel and second count (median weeks, IQR)	6 (6–6)		6 (6–6)		6 (6–6)			6 (6–6)		6 (6–6)		
Circulating tumor cells (median, IQR)	8 (1–44)		6 (0–27)		15 (2–123)		0.025^a^	10 (1–79)		29 (2–125)		0.45^a^
CTCs > 0	76	76%	30	71%	36	80%	0.45^b^	18	78%	18	82%	1.00^b^
CTCs ≥ 5	54	54%	22	52%	28	62%	0.39^b^	14	61%	14	64%	0.73^b^

a Mann–Whitney *U*‐test.

b Fisher's exact test.

Bold values indicate significant values.

Consistent with our previous studies reporting on a subset of this cohort (Onstenk *et al.*, 2015; Sieuwerts *et al.*, 2018), *AR‐V7* was detected in 48% (25 out of 52) of the evaluable baseline samples. Of the 12 patients previously treated with abiraterone or enzalutamide, 6 (50%) expressed *AR‐V7* at baseline compared with 19/40 patients (48%) who had not received prior abiraterone or enzalutamide (*P* = 1.00, Table 1, right columns).

### 3.2 Prevalence of the *AR‐Vs*

Ninety‐nine samples were evaluable for the splice‐variant analyses; 52 were baseline samples and 47 were follow‐up samples taken after the second cycle of cabazitaxel. Evaluable sample pairs to assess changes in *AR‐V* status during cabazitaxel were available for 26 patients.

The prevalence of full‐length *AR* and the four *AR‐Vs* in the baseline and follow‐up samples is shown in Table 2. Whereas 98% of the baseline patients were positive for *AR‐FL*, the detection rates for *AR‐V1, AR‐V3, AR*‐*V7,* and *AR‐V9* were lower at 31%, 31%, 48%, and 42%, respectively. Twenty‐four of the 36 (67%) patients positive for one *AR‐V* co‐expressed at least one other *AR‐V*. After two cycles of cabazitaxel, 96% of the patients remained positive for *AR‐FL*, the variants *AR‐V1, AR‐V3, AR*‐*V7,* and *AR‐V9* were detected in 30%, 19%, 40%, and 32% of the patients, respectively. Twenty‐six of the 36 patients positive for one *AR‐V* co‐expressed at least one other *AR‐V* (50% of all patients, Table 2, right panel; Table S5). Except for *AR‐V9*, loss of expression during cabazitaxel treatment was more frequently observed than a gain, albeit not statistically significant (Table 2, bottom panel).

**Table 2:** Prevalence of *AR‐FL* and the four *AR* splice variants at base line and follow‐up. Detection of different splice variants in the evaluable samples at baseline (upper panel) and after the second cabazitaxel cycle (lower row). The right panel shows the presence of multiple *AR‐V* in one sample at a specific time point. The lower panel shows the changes in positivity for the different *AR‐Vs* during cabazitaxel treatment; no significant differences in the directions of changes were observed. Percentages in the cross tables may not add up to exactly 100% due to rounding.

	*N*	*AR‐FL*		*AR‐V1*+		*AR‐V3*+		*AR‐V7*+		*AR‐V9*+		Number of patients with positive *AR‐Vs*									
												0		1		2		3		4	
Positive at baseline	52	51	98%	16	31%	16	31%	25	48%	22	42%	16	31%	12	23%	10	19%	9	17%	5	10%
Positive after c2	47	45	96%	14	30%	9	19%	19	40%	15	32%	21	45%	7	15%	10	21%	6	13%	3	6%
Overlapping baseline vs c2	26																				
Remain pos		25	96%	6	23%	3	12%	8	31%	10	38%										
Pos ‐‐> Neg		1	4%	5	19%	9	35%	6	23%	1	4%										
Neg ‐‐> Pos		0	0%	3	12%	2	8%	4	15%	5	19%										
Remain neg		0	0%	12	46%	12	46%	8	31%	10	38%										
McNemar for change during treatment		1.00		0.73		0.06		0.75		0.22											

c2; after the 2nd cycle of cabazitaxel.

### 3.3 CTC count and *AR‐Vs*

No statistically significant association (*P* < 0.01) was observed between *AR‐Vs* and any of the baseline clinical parameters, including prior anti‐AR‐targeted treatment (Table S6). After correction for sufficient epithelial signal, only patients positive for either *AR‐V1* or *AR‐V9* had higher median baseline CTC counts as measured in parallel from the enumeration tube. Only patients positive for *AR‐V3* also had higher median CTC counts after the second cycle of cabazitaxel.

The correlations in ddC_q_ values between the *AR‐Vs*, *AR‐FL,* and the CTC counts are shown in Table 3. Noteworthy, both at baseline and at follow‐up, there were no significant correlations between *AR‐V3* and *‐V7* and CTC count*.* At baseline, only *AR‐V1* correlated with CTC count, and in the on‐treatment samples, this was only the case for *AR‐FL* and *AR‐V9*. Furthermore, at baseline only *AR‐V3* correlated with *AR‐FL*, while *AR‐V7* correlated with the presence of *AR‐V9*. Interestingly, the strength of the observed correlations between *AR‐V7* and the other splice variants was stronger in the on‐treatment blood samples.

**Table 3:** Correlations between CTC count, *AR-FL* and the *AR-Vs.* Spearman correlation coefficients and corresponding *P *values from the correlations between the CTC count, *AR‐FL,* and the four *AR‐Vs* at baseline (top panel) and during follow‐up after the second cycle of cabazitaxel chemotherapy (bottom panel).

	Baseline [*N* = 50^a^/ *N* = 52]											
	CTC count^a^		*AR‐FL*		*AR‐V1*		*AR‐V3*		*AR‐V7*		*AR‐V9*	
	*r*_s_	*P*	*r*_s_	*P*	*r*_s`_	*P*	*r*_s_	*P*	*r*_s_	*P*	*r*_s_	*P*
*AR‐FL*	0.03	0.84										
*AR‐V1*	0.43	**< 0.01**	0.23	0.11								
*AR‐V3*	0.24	0.10	0.36	**< 0.01**	0.31	0.02						
*AR‐V7*	0.01	0.96	0.14	0.33	0.00	0.99	0.06	0.69				
*AR‐V9*	0.29	0.04	0.11	0.45	0.30	0.03	0.22	0.11	0.45	**< 0.01**		

	Follow‐up [*N* = 47]											
	CTCcount		*AR‐FL*		*AR‐V1*		*AR‐V3*		*AR‐V7*		*AR‐V9*	
	*r*_s_	*P*	*r*_s_	*P*	*r*_s_	*P*	*r*_s_	*P*	*r*_s_	*P*	*r*_s_	*P*
*AR‐FL*	**0.42**	**< 0.01**										
*AR‐V1*	0.37	0.01	0.36	0.01								
*AR‐V3*	0.10	0.49	0.29	0.05	0.16	0.29						
*AR‐V7*	0.35	0.02	**0.50**	**< 0.01**	**0.62**	**< 0.01**	**0.38**	**< 0.01**				
*AR‐V9*	**0.47**	**< 0.01**	0.30	0.04	0.33	0.02	0.14	0.35	**0.44**	**< 0.01**		

Spearman rank correlation test; 2‐sided *P*‐values < 0.01 were considered statistically significant.

a For 50 of the *n* = 52 patients, a CTC count was available.

Bold values indicate significant values.

### 3.4 Association with CTC and PSA response

First, we evaluated the CTC5‐ and CTC0‐RR after two cycles of cabazitaxel (defined as a conversion from ≥ 5 CTCs/7.5 mL to < 5 CTCs or a clearance from ≥ 1 CTC(s) to 0 during treatment, respectively). The overall CTC5‐RR in the 43 evaluable patients was 35%; the overall CTC0‐RR for these patients was 16%. No significant differences at our multiple correction adjusted *P* < 0.01 were seen in both CTC‐RRs by *AR‐FL*, *AR‐V1, AR‐V3,* and *AR‐V7* status (Table 4). Only patients with *AR‐V9‐*positive CTCs at baseline less frequently achieved a CTC5‐RR response, associating the presence of *AR‐V9*‐positive CTCs at baseline with poor outcome to cabazitaxel (Table 4, upper panel). Next, we evaluated the PSA response. None of the splice variants associated with PSA response as measured at 12 weeks (Table 4, lower panel).

**Table 4:** *AR‐Vs* correlated with response. Cross‐tables between the presence of the *AR‐Vs* at baseline and the observed CTC5‐RR, CTC0‐RR and PSA responses. Overall response rates are shown in the first column. The response rates by *AR‐V *status are shown as percentages of the total per *AR‐V* positive and *AR‐V *negative subgroups and differences were tested by Fisher exact test for the CTC5 and CTC0‐RR and the ordinal χ2 for the PSA‐RRs. Percentages in the cross tables may not add up to exactly 100% due to rounding.

	All patients		*AR‐FL*				*AR‐V1*				*AR‐V3*				*AR‐V7*				*AR‐V9*			
			Negative		Positive		Negative		Positive		Negative		Positive		Negative		Positive		Negative		Positive	
CTC response, *N*	43		1	100%	42	100%	28	100%	15	100%	28	100%	15	100%	22	100%	21	100%	24	100%	19	100%
CTC5‐RR; Yes	15	35%	0	0%	15	36%	13	46%	2	13%	13	46%	2	13%	8	36%	7	33%	13	54%	2	11%
CTC5‐RR; No	28	65%	1	100%	27	64%	15	54%	13	87%	15	54%	13	87%	14	64%	14	67%	11	46%	17	89%
χ^2^ yes vs no	N/A		1.00				0.045								0.045				**0.004**			
CTC0‐RR; Yes	7	16%	0	0%	7	17%	5	18%	2	13%	7	25%	0	0%	4	18%	3	14%	5	21%	2	11%
CTC0‐RR; No	36	84%	1	100%	35	83%	23	82%	13	87%	21	75%	15	100%	18	82%	18	86%	19	79%	17	89%
χ^2^ yes vs no	N/A		1.00				1.00				0.08				1.00				0.44			
PSA response at 12 weeks, *N*	52	100%	1	100%	51	100%	36	100%	16	100%	36	100%	16	100%	27	100%	25	100%	30	100%	22	100%
No	33	63%	1	100%	32	63%	23	64%	10	63%	23	64%	10	63%	15	56%	18	72%	17	57%	16	73%
≥30–50%	4	8%	0	0%	4	8%	1	3%	3	19%	2	6%	2	13%	2	7%	2	8%	1	3%	3	14%
≥50%	15	29%	0	0%	15	29%	12	33%	3	19%	11	31%	4	25%	10	37%	5	20%	12	40%	3	14%
χ^2^	N/A		1.00				0.12				0.72				0.34				0.08			
χ^2^ ≥ 30% vs < 30%	N/A		1.00				1.00				1.00				0.26				0.26			

CTC5‐RR; a conversion from ≥5 CTCs/7.5 mL to <5 CTCs during treatment

CTC0‐RR; a clearance from ≥1 CTC(s) to 0 during treatment

*P* values are from 2‐tailed Fisher's exact tests. Percentages may not add up to 100% due to rounding.

Bold values indicate significant values.

### 3.5 Association with overall survival

Lastly, we investigated the impact on OS of the presence of the *AR‐Vs,* at baseline and after two cabazitaxel cycles, respectively. Median OS for the *n* = 52 baseline patients was 8.3 months (95% CI: 7.1–13.0) and 7.7 (95% CI: 7.0–10.6) for the *n* = 47 patients at follow‐up.

At baseline, the *AR‐V1* status and CTC count associated with decreased OS in the univariate model. Multivariate Cox regression analyses showed that at baseline only CTC count remained an independent poor prognostic factor for OS (Table 5, upper panel).

**Table 5:** *AR‐Vs* correlated with overall survival. Univariate (left columns) and multivariate (right columns) Cox regression analyses for the associations of baseline parameters (upper rows) and on‐study parameters (lower rows) with OS. For the on‐study parameters, survival time was calculated from the second blood draw. The multivariate Cox regression analysis was carried out using backward selection. Non‐significant factors were removed stepwise and the HRs and *P *values were taken from the removal step.

	Univariate			Multivariate		
	HR	95% CI	*P*	HR	95% CI	*P*
Baseline (*N* of cases = 52)						
*AR‐FL*, continuous	1.0	0.9–1.1	0.90			
*AR‐V1*, positive vs negative	**1.9**	**1.0**–**3.6**	**0.04**	1.3	0.7–2.6	0.42
*AR‐V3*, positive vs negative	1.9	1.0–3.5	0.06			
*AR‐V7*, positive vs negative	1.2	0.7–2.1	0.57			
*AR‐V9*, positive vs negative	1.5	0.8–2.8	0.17			
CTC count, ≥5 vs < 5 CTCs	**4.8**	**2.0**–**11.4**	**0.001**	**4.3**	**1.8**–**10.6**	**0.001**
PSA, continuous	1.0	1.0–1.0	0.59			
Anti‐AR prior to cabazitaxel, yes vs no	1.4	0.8–2.6	0.26			
After two cabazitaxel cycles (*N* of cases = 47)						
*AR‐FL*, continuous	**1.2**	**1.0**–**1.3**	**0.005**	1.1	0.9–1.2	0.30
*AR‐V1*, positive vs negative	**3.0**	**1.5**–**6.3**	**0.003**	**2.4**	**1.1**–**5.1**	**0.031**
*AR‐V3*, positive vs negative	1.7	0.8–3.7	0.16			
*AR‐V7*, positive vs negative	1.8	0.9–3.4	0.08			
*AR‐V9*, positive vs negative	1.6	0.8–3.2	0.19			
CTC count, ≥5 vs < 5 CTCs	**3.5**	**1.6**–**7.4**	**0.001**	**2.4**	**1.0**–**5.9**	**0.047**
PSA at 12 weeks, continuous	1.0	1.0–1.0	0.17			
Anti‐AR after cabazitaxel, yes vs no	**0.4**	**0.2**–**0.8**	**0.009**	0.6	0.3–1.1	0.11

Bold values indicate significant values.

3.6

**Figure 2:**
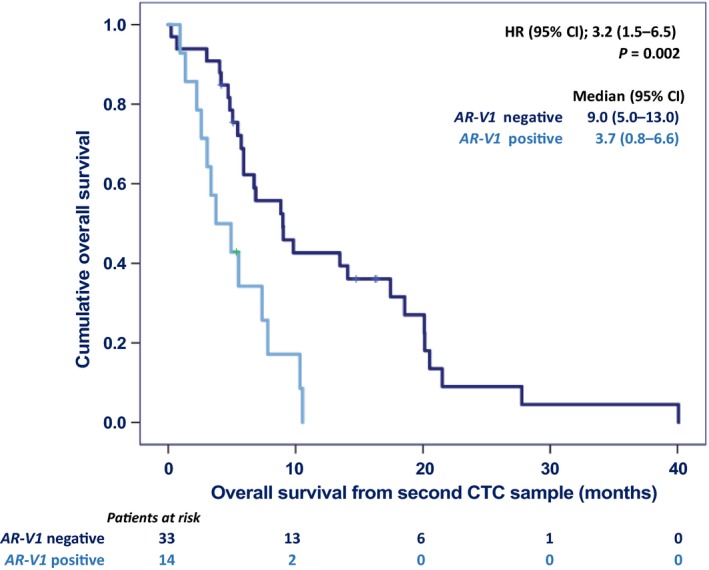
Overall survival as a function of *AR‐V1* in circulating tumor cells after two cycles of cabazitaxel. The reported *P‐*value is from a log‐rank test and the test statistics from Cox regression analyses.

*AR‐FL*, *AR‐V1,* and CTC count measured after two cycles of cabazitaxel, and anti‐AR treatment after cabazitaxel, associated with OS in the univariate model. In the multivariate model, CTC count remained an independent prognostic factor. *AR‐V1* status overruled *AR‐FL* and was an additional CTC5‐independent predictive factor for OS after two cycles of cabazitaxel (Table 5, bottom panel). The OS curves as a function of *AR‐V1* in CTCs after two cycles of cabazitaxel are shown in Fig. 2.

## 4 Discussion

Whereas the presence of *AR‐FL* and the constitutively ligand independent active *AR‐V3* and *AR‐V7* were not associated with outcome to therapy, the presence of the conditionally activated *AR‐V9* and *AR‐V1* associated with a negative CTC RR during treatment and decreased OS after two cycles of cabazitaxel, respectively. In this study, we demonstrated in an extended cohort of metastatic prostate cancer patients that the presence of *AR‐V7* in CellSearch‐enriched CTCs is not associated with outcome to cabazitaxel. In line with our previous findings in the first 29 patients with ≥ 10 CTCs we studied at baseline (Onstenk *et al.*, 2015), we detected *AR‐V7* at baseline in 48% of all the 52 evaluable patients. We did not find a correlation between the presence of *AR‐V7* at baseline and prior anti‐AR‐targeted therapy. After the second cycle of cabazitaxel, the prevalence of *AR‐V7* had slightly but not significantly decreased from 48% to 40%, indicating that expression of *AR‐V7* was neither specifically lost nor gained during treatment with cabazitaxel.

To investigate whether other *AR* splice variants than *AR‐V7* were associated with response or outcome to cabazitaxel, we similarly analyzed the expression levels of another variant with constitutive activity (De Laere *et al.*, 2017; Jernberg *et al.*, 2017) (*AR‐V3*) and two more cell–context‐dependent variants (*AR‐V1* and *AR‐V9*) (Hu *et al.*, 2009; Hu *et al.*, 2011; Jernberg *et al.*, 2017) in CTCs of mCRPC patients at baseline and after 2 cycles of cabazitaxel.

Perhaps surprisingly, taking our associations with outcome to cabazitaxel only associated with the conditionally activated *AR‐V1* and *AR‐V9* into account, we noticed a positive correlation between *AR‐V7* and *AR‐V9* at baseline and *AR‐V7* and all studied splice variants after two cycles of cabazitaxel. However, although the presence of *AR‐V1, AR‐V3*, *AR‐V7,* and *AR‐V9* in CTCs at baseline and during cabazitaxel treatment was frequently observed in our study, only 10% of the patients co‐expressed all four *AR‐Vs* at baseline and 6.5% after two cycles of cabazitaxel, suggesting the presence of an *AR* splice variant at our indicated cutoffs is a splice‐variant‐specific event.

Importantly, concern has been shared that the detection of *AR* splice variants might be intrinsically related to CTC count. However, after using our cutoffs and adjusting for the epithelial content present in the blood samples, at baseline only *AR‐V1* and at follow‐up only *AR‐FL* and *AR‐V9* correlated significantly with CTC count.

Confirming our prior observations (Onstenk *et al.*, 2015), we found no associations between the *AR‐V7* status and outcome to cabazitaxel in terms of the CTC/PSA‐RR and/or OS. Similarly, the other constitutively activated AR variant we investigated, *AR‐V3*, had no association with these outcome measures. In contrast, the presence of the conditionally activated *AR‐V1* showed an association with OS at baseline and after two cycles of cabazitaxel; patients with positive CTCs for the other conditional variant, *AR‐V9*, associated with CTC5 response to cabazitaxel, but we found no impact of the presence of *AR‐V9* on OS. A lower sensitivity of our assay to detect *AR‐V9* could explain the lack of an association (although the LODs) were with the equivalent of 0.11 cells comparable for the two assays, the LOQ was the equivalent of 0.02 cells for *AR‐V1* and 12 cells for *AR‐V9*; see also Table S2). Interestingly, the presence of *AR‐V1* in CTCs was a poor prognostic factor for OS at baseline, but this might merely have been a reflection of CTC count. After the second cabazitaxel cycle, the presence of *AR‐V1* kept its prognostic value. And at this time point, the presence of *AR‐V1* was, together with high CTC count, an independent factor for worse OS in our multivariate analysis.

The results from our study compare well with recent reports on the clinical value of the *AR‐V7* status of CTCs. The observed *AR‐V7* positivity rate of 48% at baseline in this study is similar to the previously reported 18–46% (Antonarakis *et al.*, 2015; Antonarakis *et al.*, 2014; De Laere *et al.*, 2017; Miyamoto *et al.*, 2015; Scher *et al.*, 2016). The lack of association between the *AR‐V7* status of CTCs and outcome to taxane‐based chemotherapy confirms both our prior findings (Onstenk *et al.*, 2015), as well as those of others (Antonarakis *et al.*, 2015; Scher *et al.*, 2018). On the contrary, an association has been reported between the presence of *AR‐V7* at the protein level and worse outcome to taxane treatment, especially when the protein was localized in the cell nucleus (Scher *et al.*, 2017; Scher *et al.*, 2016; Tagawa *et al.*, 2019). Nonetheless, the negative impact on outcome to anti‐AR treatment was with an hazard ratio (HR) of 10.4 much higher than the HR of 3.2 for taxane treatment (Scher *et al.*, 2017; Scher *et al.*, 2016). The possible differences in functionality and clinical relevance of overall mRNA levels vs translated protein levels of *AR‐V7* need further investigation.

In line with our observations, switches in the *AR‐V7* status of CTCs during treatment have been reported. In a small group of just 14 patients, a loss of *AR‐V7* only occurred during taxane treatment and not during anti‐AR treatment (Nakazawa *et al.*, 2015). We found 6 of 14 patients (43%) with *AR‐V7*‐positive CTCs at baseline who reverted to *AR‐V7* negative during cabazitaxel (Table 2). Similar high percentages were seen for the other constitutively active *AR‐V3* (75%). For the conditionally active *AR‐V1* and *AR‐V9,* these fractions were less with 45% and 9%, respectively. Given the negative impact of the presence of *AR‐V7* on outcome to anti‐AR‐targeted compounds (Antonarakis *et al.*, 2015; Antonarakis *et al.*, 2014; Scher *et al.*, 2016), one may hypothesize that a loss of *AR‐V7* and perhaps *AR‐V3* during chemotherapy translates to a regained sensitivity to anti‐AR treatments. However, this hypothesis needs to be tested.

To the best of our knowledge, only two studies reported on the clinical relevance of the presence of other *AR‐Vs* besides *AR‐V7* in the CTCs of patients with mCRPC (De Laere *et al.*, 2017; De Laere *et al.*, 2019). In the first study (De Laere *et al.*, 2017), characterization of circulating tumor DNA and CTCs from 30 mCRPC patients showed a correlation between the presence of structural variants in the *AR* gene and *AR‐Vs*. In the second, larger study of the same group (De Laere *et al.*, 2019), the prognostic value of different *AR‐V*s prior to starting first‐line abiraterone and enzalutamide treatment was investigated and demonstrated that *TP53* inactivation was an independently associated negative response biomarker, while *AR* and its splice variants were not. The authors concluded that further comprehensive AR profiling studies are required to determine which patients have a relevant *AR* biomarker output. Besides, preclinical studies show evidence for dimerization and interactions between different *AR‐Vs*, such as *AR‐V7* and *AR‐V1 *(Zhan *et al.*, 2017), as well as cross‐reactivity of assays between different *AR‐Vs*, such as *AR‐V7* and *AR‐V9 *(Kohli *et al.*, 2017). Mutual correlations and individual biological functions of the different *AR‐Vs* as well as assay specificity will thus need to be studied in more detail and will be the subject of a planned future study from our group.

A major advantage of our CellSearch‐based assay to measure the expression of a panel of *AR‐Vs* and *AR‐FL* in CTCs as opposed to assays like the AdnaTest (Qiagen) is that it provides the possibility to obtain a CTC count in parallel to the CTC characterization. Importantly, our qPCR‐based characterization assay incorporates a correction for the number of CTCs present in a sample by use of the expression levels of epithelial genes to limit confounding of the analyses by the CTC count. Both the CTC count and CTC dynamics during treatment have robustly been shown to be strongly associated with outcome (de Bono *et al.*, 2008; Scher *et al.*, 2015; Scher *et al.*, 2009).

The recruitment of patients from a clinical trial provided us with a homogeneous group of patients and samples with corresponding, prospectively collected, clinical data. Only patients with sufficient epithelial input in the sample were selected for the analyses to ascertain reliable results from the *AR‐V* assays. A higher median baseline CTC count of 83 vs seven CTCs/7.5 mL blood was detected in the evaluable patients vs the excluded patients with an epithelial signal too low for a reliable *AR‐V* assessment (Sieuwerts *et al.*, 2018), respectively, which supports the robustness of our assay in measuring tumor‐driven signals.

The main limitation of this prospective study is the modest sample size. No formal sample size calculations were performed and the exclusion of 50% of the patients based on insufficient epithelial signal in the samples for a meaningful *AR‐V* analysis limits the power of our analyses. To diminish the chance of type I errors, we applied multiple testing corrections by bootstrapping and employing a more stringent *P* value < 0.01. Validation of our results in other retrospective and prospective studies is warranted. Other blood‐based assays, for example on exosomes (Del Re *et al.*, 2017) or whole blood (Liu *et al.*, 2016; Qu *et al.*, 2017), may in this context aid in reporting the *AR‐V* status in a larger proportion of patients, including those without or with low number of CTCs.

## 5 Conclusions

In conclusion, we confirm that the presence of *AR‐V7* has no impact on outcome to cabazitaxel treatment in patients with mCRPC. We found that the presence of other *AR‐Vs,* in particular *AR‐V1* and *AR‐V9,* though, may predict adverse outcome in terms of survival after cabazitaxel and CTC‐RR during treatment, respectively. Especially our analysis showing that the CTC‐adjusted *AR‐V1* detection after two cycles of cabazitaxel was an independent prognostic factor for OS is a potentially promising finding. Future studies on the functionality and clinical relevance of different *AR‐Vs* are currently being set up in order to increase our knowledge and understanding. A prospective clinical study on the predictive value of the *AR‐V7* status of CellSearch‐enriched CTCs is currently ongoing (NCT03050866).

## Conflict of interest

The described *AR‐V7* assay has been patented (#WO2016133387 A1) by WO, AMS, and SS.

## Author contributions

AMS and WO involved in manuscript preparation, design of the work, data acquisition and data analysis; JK, CMB, NMV, and BdL acquired the data; LD, PH, AB, HM, and GJC included the patient; WMW, GWJ, AJN, and AHM designed the study; JWM supervised the project: SS supervised the project and designed the study. All co‐authors have read and approved of the manuscript and acknowledge their contributions.

## Supporting information


**Table S1.** Details Taqman gene expression assays.
**Table S2.** Limit of detection and limit of quantification for the *AR‐V* assays. Experiments were performed as described before for *AR‐V7 *(Sieuwerts *et al*., 2018)*.* In brief, the limit of detection (LOD), defined as the lowest input of RNA isolated from VCaP cells that could be reliably identified as being qualitatively present in the sample, was calculated to be the RNA equivalent of 0.03 (for *AR‐WT*) to 0.5 (for *AR‐V3*) VCaP cells in the final RT–qPCR. The limit of quantification (LOQ), defined as the lowest input of RNA isolated from VCaP cells that could be reproducibly quantified, was set at a CV of less than 25% and calculated to be equal to the RNA equivalent of 0.02 (for *AR‐V1*) to 12 (for *AR‐V9*) VCaP cells in the final RT–qPCR.
**Table S3.** Intralaboratory validation for the *AR‐V* assays on paired clinical samples. Experiments were performed as described before for *AR‐V7 *(Sieuwerts *et al*., 2018)*.*
**Table S4.** Interlaboratory validation for the *AR‐V* assays. Experiments were performed as described before for *AR‐V7 *(Sieuwerts *et al*., 2018)*.*
**Table S5.** Patient characteristics of all 124 patients participating to the CTC study as shown in Fig. 1.
**Table S6.** Extension of Table 1, now also including comparisons between the patient groups with CTCs negative or positive for the other *AR‐Vs* besides *AR‐V7*.

**Data S1.** In‐ and exclusion criteria for participation in CABARESC trial.
