# Supplementary material for: AR splice variants in circulating tumor cells of patients with castration‐resistant prostate cancer: relation with outcome to cabazitaxel
Source: Mol Oncol. 2019 Jun 28;13(8):1795–807. doi: 10.1002/1878-0261.12529 (PMC6670012; doi:10.1002/1878-0261.12529)
Supplement: Supplementary file 2 — Data S1. In‐ and exclusion criteria for participation in CABARESC trial. [file MOL2-13-1795-s002.docx]

### Supplementary data 1.

#### In- and exclusion criteria for participation in CABARESC trial

Inclusion criteria:

- Metastatic castrate resistant prostate cancer with documented disease progression, defined as:
  - Rising PSA levels; at least two consecutive rises over a reference value and at least one week apart, or a PSA rise of ≥2.0 μg/L
  - Appearance of new lesions or documented disease progression on a CT scan or bone scan
- Previous treatment with docetaxel
- Age ≥18 years
- WHO performance status ≤1
- Adequate renal function (serum creatinine ≤1.5 x upper limit of normal (ULN) and/or MDRD calculated creatinine clearance ≥50 mL/min) and hepatic function (total bilirubin ≤1.0 x ULN, alanine aminotransferase and aspartate aminotransferase ≤2.5 x ULN, or in case of liver metastases ≤5 x ULN, and alkaline phosphatase < 5 x ULN, or in case of bone metastases <10 x ULN), within 21 days before randomization
- Adequate hematological blood counts (absolute neutrophil count ≥1.5 x 10^9^/L and platelets ≥100 x 10^9^/L) within 21 days before randomization
- Castration, either surgically or by continued LHRH agonist therapy
- Written informed consent according to ICH-GCP

Exclusion criteria:

- Impossibility or unwillingness to take oral drugs
- Impossibility to start cabazitaxel treatment at a dose of 25 mg/m^2^
- Serious illness or medical unstable conditions requiring treatment, symptomatic central nervous system metastases or history of a psychiatric disorder that would hinder the understanding and obtaining of informed consent
- Use of medications or dietary supplements known to induce or inhibit CYP3A
- Use of hormonal agents other than GnRH agonists
- Known hypersensitivity to corticosteroids
- Any active systemic or local bacterial, viral, or fungal infection
- Ulcerative colitis, Crohn’s disease, or celiac disease (active or in medical history)
- Ostomy
- Planned/active simultaneous yellow fever vaccine
- Geographical, psychological, or other non-medical conditions interfering with follow-up.
