# Supplementary material for: AR splice variants in circulating tumor cells of patients with castration‐resistant prostate cancer: relation with outcome to cabazitaxel
Source: Mol Oncol. 2019 Jun 28;13(8):1795–807. doi: 10.1002/1878-0261.12529 (PMC6670012; doi:10.1002/1878-0261.12529)
Supplement: Supplementary file 3 [file MOL2-13-1795-s003.docx]

## Supplementary Tables

**Supplementary Table 1:** Details Taqman gene expression assays.

**Supplementary Table 2:** Limit of detection and limit of quantification for the *AR-V* assays. Experiments were performed as described before for *AR-V7*^1^*.*

**Supplementary Table 3:** Intra-laboratory validation for the *AR-V* assays on paired clinical samples. Experiments were performed as described before for *AR-V7*^1^*.*

**Supplementary Table 4:** Inter-laboratory validation for the *AR-V* assays. Experiments were performed as described before for *AR-V7*^1^*.*

**Supplementary Table 5:** patient characteristics of all 124 patients participating to the CTC study as shown in **Supplementary Figure 1**. Column D indicates the patients that have also been included in the first analyses for our prior report^2^. Column P shows whether the patient has been included in the current analyses and if not, for what reason (Q). The columns R-AJ show sample characteristics for the baseline (R-X) and, if appropriate, the follow-up blood samples (Z-AJ), including the strength of the *AR-FL* signal, and the presence of the four *AR-Vs*.

**Supplementary Table 6:** extension of table 1, now also including comparisons between the patient groups with CTCs negative or positive for the other *AR-Vs* besides *AR-V7*. The first columns show the patient characteristics of all patients with ≥1 blood sample (*N* = 118 from the total of 124, first column), evaluable patients for the analyses with a baseline blood sample containing sufficient reference and epithelial gene signal (*N* = 52, third column) and non-evaluable patients with insufficient epithelial signal, indicating the absence of tumor cell signal (*N* = 52, second column); *P* values in the fourth column are from the comparisons between the evaluable and excluded patients. To the right are the comparisons between evaluable patients with *AR-V1/3/7/9* negative versus positive CTCs at baseline with corresponding *P* values from independent samples *t* tests (for age), non-parametric Mann-Whitney U test (for baseline chemistry and CTC counts) and χ^2^ or Fisher exact tests for the categorical variables.

1. Sieuwerts AM, Mostert B, van der Vlugt-Daane M, Kraan J, Beaufort CM, Van M, Prager WJC, De Laere B, Beije N, Hamberg P, Westgeest HM, Tascilar M, et al. An In-Depth Evaluation of the Validity and Logistics Surrounding the Testing of AR-V7 mRNA Expression in Circulating Tumor Cells. *J Mol Diagn* 2018;**20**: 316-25.

2. Onstenk W, Sieuwerts AM, Kraan J, Van M, Nieuweboer AJ, Mathijssen RH, Hamberg P, Meulenbeld HJ, De Laere B, Dirix LY, van Soest RJ, Lolkema MP, et al. Efficacy of Cabazitaxel in Castration-resistant Prostate Cancer Is Independent of the Presence of AR-V7 in Circulating Tumor Cells. *European urology* 2015;**68**: 939-45.
